# PON-Tm: A Sequence-Based Method for Prediction of Missense Mutation Effects on Protein Thermal Stability Changes

**DOI:** 10.3390/ijms25158379

**Published:** 2024-07-31

**Authors:** Jiahao Kuang, Zhihong Zhao, Yang Yang, Wenying Yan

**Affiliations:** 1 Center for Systems Biology, School of Basic Medical Sciences, Suzhou Medical College of Soochow University, Suzhou 215123, China; 2 School of Computer Science & Technology, Soochow University, Suzhou 215000, China; 3 Computing Science and Artificial Intelligence College, Suzhou City University, Suzhou 215004, China; 4 Institute of Intelligent Software and Data Engineering, Suzhou City University, Suzhou 215004, China; 5 Jiangsu Province Engineering Research Center of Precision Diagnostics and Therapeutics Development, Suzhou 215123, China

**Keywords:** protein thermal stability, mutation, protein embedding

## Abstract

Proteins, as crucial macromolecules performing diverse biological roles, are central to numerous biological processes. The ability to predict changes in protein thermal stability due to mutations is vital for both biomedical research and industrial applications. However, existing experimental methods are often costly and labor-intensive, while structure-based prediction methods demand significant computational resources. In this study, we introduce PON-Tm, a novel sequence-based method for predicting mutation-induced thermal stability variations in proteins. PON-Tm not only incorporates features predicted by a protein language model from protein sequences but also considers environmental factors such as pH and the thermostability of the wild-type protein. To evaluate the effectiveness of PON-Tm, we compared its performance to four well-established methods, and PON-Tm exhibited superior predictive capabilities. Furthermore, to facilitate easy access and utilization, we have developed a web server.

## 1. Introduction

Protein thermostability refers to proteins’ resilience in maintaining their three-dimensional conformations when exposed to elevated temperatures [1]. When subjected to temperatures outside their optimal range, proteins can undergo denaturation, ultimately leading to the loss of their native structural integrity and, consequently, their functional capabilities. Therefore, the enhancement of protein thermostability has become a critical focal point in the fields of biopharmaceuticals, enzyme engineering, and food engineering research [2,3,4]. In this regard, single-point mutation plays a significant role in protein engineering. By altering a single amino acid residue within the protein sequence, the mutation can confer enhanced stability and activity to a protein, allowing it to function effectively at temperatures exceeding its normal physiological range [5,6].

In recent decades, numerous computational methodologies have been developed to evaluate the stability changes of purified and intracellular proteins, effectively mitigating the exorbitant costs and addressing the scarcity of available data [7,8,9,10]. Current methodologies can be broadly categorized into two main groups based on the target proteins. The first group, represented by AUTOMUTE [8] and HotMuSiC, is universally applicable to all protein types. These methods leverage a diverse set of features, such as physicochemical properties, secondary structure, solvent accessibility, conservation scores, position-specific scoring matrices, and temperature-dependent statistical potentials, to forecast thermostability variations. The second group, exemplified by MPTherm-pred [10], is specifically tailored to certain protein classes, incorporating distinct features inherent to those proteins for enhanced prediction accuracy. Notably, MPTherm-pred specializes in membrane proteins, frequently yielding predictions that exceed the precision of general-purpose predictors. Previous machine learning (ML) methods have relied primarily on the experimental structure of the target protein, often sourced from the Protein Data Bank (PDB), as input. However, it is worth noting that only a relatively small proportion of proteins have had their structures determined [11]. Obtaining these structures typically requires time- and resource-intensive experimental methods like X-ray crystallography or NMR.

**Figure 1:**
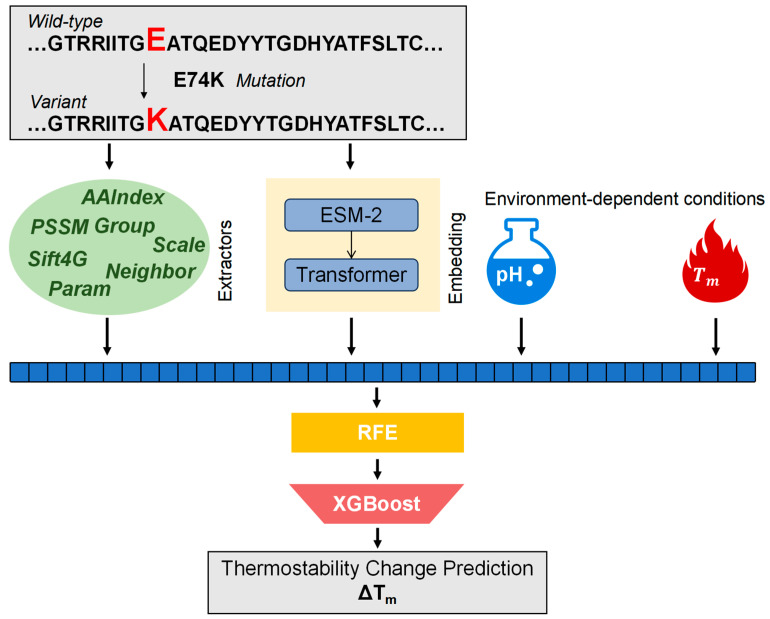
Schematic overview of PON-Tm construction.

In our present study, we present PON-Tm, an innovative sequence-based methodology for predicting thermal stability changes (ΔTm) caused by mutation. PON-Tm not only incorporates features forecasted by protein language models (PLMs) [12,13,14] from protein sequences, but also takes into account environment-dependent conditions such as pH and wild-type thermostability (Tm) (Figure 1). This integration aims to enhance the predictive accuracy of missense mutation impacts on protein thermal stability. To assess the efficacy of PON-Tm, we benchmarked its performance against four established methods. We have developed a web server for PON-Tm, which is freely available at https://www.yanglab-mi.org.cn/Pon-Tm (accessed on 28 July 2024).

## 2. Results

### 2.1. Environment-Dependent Dataset

We systematically organized the collected mutations based on different environment-dependent conditions and constructed the MutDB dataset by integrating data from ProThermDB [15], HoTMuSiC [16,17], and MPTherm [18]. To explore the impact of wild-type melting temperature (Tm) and pH on prediction accuracy, MutDB was categorized into three sub-datasets: Common (without considering any auxiliary factors), Tm (solely concentrating on the thermal stability of wild-type proteins), and pH-Tm (examining both pH and Tm conditions simultaneously). The results are summarized in Table 1. Subsequently, we conducted the training and testing of PON-Tm across all three sub-datasets within MutDB.

### 2.2. Impact of Environment-Dependent Factors on the Prediction of Thermal Stability Changes

Prior prediction methods have predominantly relied on protein and mutation data to forecast changes in thermal stability, frequently overlooking the influence of environmental conditions on thermal stability changes by adopting an averaging approach across different conditions for a single protein. However, environment-dependent factors are also crucial determinants of thermal stability [19,20]. Generally, the same protein may exhibit different Tm values under different environmental conditions. This leads to the phenomenon that the same mutation in the same protein may result in different changes in ΔTm under different environments. As exemplified in the MutDB dataset, the same protein and mutation can exhibit distinct ΔTm values under varying pH or initial Tm conditions. For instance as shown in Table 2, in the case of Iso-1 cytochrome c protein (P00044) carrying the K79I mutation, a gradual increase in pH from 3.0 to 5.0 in increments of 0.5 led to a corresponding elevation in Tm from 22.3 to 47.7, with concurrent irregular ΔTm fluctuations in thermal denaturation experiments [21].

In this study, we conducted a novel exploration of how environmental factors, particularly Tm and pH, affect ΔTm prediction—an aspect that has garnered limited attention in prior research. To achieve this, we compared the predictive performance of three distinct models: one that considers Tm, another that integrates both Tm and pH, and a third that relies solely on basic protein and mutation information without any additional inputs. As evidenced in Table 3, the model that incorporated both pH and Tm demonstrated superior predictive capabilities, achieving an impressive mean absolute error (MAE) of 2.030, root mean square error (RMSE) of 3.602, coefficient of determination (R^2^) of 0.774, and a Pearson correlation coefficient (PCC) of 0.881. The model that only factored in Tm provided slightly less accurate results. Conversely, the traditional approach, devoid of any additional environmental inputs, proved significantly less efficient, with an MAE of 4.312, RMSE of 7.068, R^2^ of 0.271, and a PCC of 0.563. Our findings underscore the critical role of environmental conditions, such as pH and Tm, in refining ΔTm predictions, paving the way for a more accurate understanding of protein stability alterations induced by mutations.

### 2.3. Deep Representation of Protein Sequence Information Using Embedding

By analogy, we conceive of amino acids in protein sequences as equivalent to letters in natural language, enabling the application of transfer learning techniques to proteins. This approach facilitates a deeper understanding of protein sequence characteristics. Therefore, in this study, we employed protein language models to carry out protein embedding, thereby extracting comprehensive features.

#### 2.3.1. Comparison of the Performance of Different Embedding Models

We utilized ProtBert [22] and two variants of the ESM-2 model [14] to embed protein sequences and subsequently evaluated and compared their performance. Detailed descriptions of these models are provided in the Section 4. Table 4 displays the performance outcomes of these three models through rigorous 10-fold cross-validation. Among the models tested, ESM-2-3B demonstrated the most outstanding performance, followed by ESM-2-650M. ProtBert, on the other hand, yielded the least impressive results. Hence, ESM-2-3B was selected as the embedding model for our final prediction.

#### 2.3.2. Comparison of the Performance of Different Context Lengths

After determining the embedding method, we progressed to selecting an optimal truncation length for the embedded sequences to maximize model performance. We evaluated six different lengths: 25, 50, 100, 200, 500, and the full sequence length. This entailed intercepting protein sequences around the mutation site to the specified lengths. When utilizing the complete protein sequence, no specific requirements are imposed on the position of mutation sites within the protein sequence. Instead, the wild-type sequence and its corresponding mutant sequence are directly employed. Table 5 presents the performance of models trained under these six length conditions, assessed through ten-fold cross-validation. Notably, MAE reached a minimum of 2.039 at an embedding length of 200, representing the best performance among all tested lengths. The RMSE, R^2^, and PCC achieved their optimal values at context lengths of 100, 500, and 500, respectively. Conversely, using the full sequence for embedding yielded the poorest performance, significantly inferior to the optimal values in all four evaluation metrics. Considering that a shorter context length reduces the sequence length requirement and mitigates the risk of overfitting, we ultimately selected a length of 200 for training the final model.

### 2.4. Feature Selection

In addition to environmental factors and features extracted from embedding models, we acquired traditional characteristics of protein sequences, including amino acid properties from AAindex (27), mutation site adjacent features, positional features, and others. In total, 3704 features were incorporated. Following this, to prevent model overfitting, feature selection was undertaken using recursive feature elimination (RFE) [23] and recursive feature elimination with cross-validation (RFECV).

In our investigation, we employed RFE on feature subsets comprising 50, 100, 200, 500, 1000, and the entire set of 3704 features. As for RFECV, we established a minimum threshold of 50 features, which led to an automatically determined optimal count of 595 features. Both RFE and RFECV underwent a rigorous 10-fold cross-validation process to ensure the robustness of our results. Detailed outcomes of this analysis are presented in Table 6. Among the results, the 200 features chosen by RFE demonstrated the most superior combined performance across four evaluation metrics. Specifically, compared to the baseline without feature selection, there were pronounced improvements in all metrics. The MAE significantly decreased from 2.093 to 1.930, and the RMSE showed a comparable improvement. Furthermore, R^2^ rose from 0.743 to 0.797, and the PCC value climbed from 0.865 to 0.893.

Table 7 enumerates the distribution of the different types of features within these 200 features. Notably, RFE selected features such as pH, Tm, and embedding. Additionally, our newly introduced Scale and Neighbor features were also chosen. Features extracted from AAindex, Pssm, and Param played a critical role as well, consistent with their widespread effectiveness demonstrated in previous studies. The importance of these 200 features is listed in Table S1.

### 2.5. Performance Comparison with Other Methods

**Figure 2:**
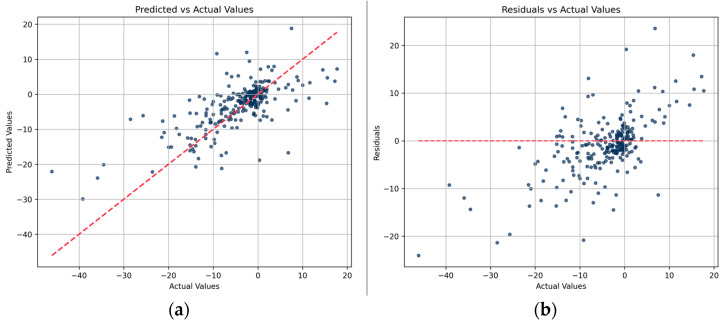
The correlation between PON-Tm prediction values and actual values on the test dataset (**a**) and between actual values and residuals obtained by subtracting the predicted values from the actual values (**b**). The red dashed line represents the reference baseline.

We finally have constructed a predictive model, designated as PON-Tm, using XGBoost and based on 200 carefully selected features. Initially, we assessed the efficacy of PON-Tm by computing the correlation between its predicted and actual values. As illustrated in Figure 2a, there is a significant correlation between the predicted and actual values, evidenced by a correlation coefficient of 0.70 and *p*-value below 5.41 × 10^−22^. Furthermore, in the plot comparing residuals against actual values (Figure 2b), the data points appear generally randomly distributed around the reference residual = 0 line.

To evaluate its performance, we compared PON-Tm with previously established tools, namely AUTO-MUTE_SVM [8], AUTO-MUTE_REPT [8], HotMuSic [16], and MPtherm-pred [10], using the test set T267 (Table 8). The results indicated that PON-Tm achieved the best performance on this test set, with an MAE of 3.18, an RMSE of 6.10, an R^2^ of 0.49, and a PCC of 0.72. Among the other predictors, AUTOMUTE and HotMuSiC demonstrated the next-best performance. Conversely, MPTherm-pred showed the weakest performance, significantly lagging behind the other four predictors in all four evaluation metrics. Notably, its R^2^ value was found to be less than 0, suggesting that the model is not adequate for characterizing the test set T267.

### 2.6. PON-Tm Web Application

The PON-Tm web application is freely accessible at https://www.yanglab-mi.org.cn/Pon-Tm/ (accessed on 28 July 2024). This program accepts protein sequences (or their corresponding UniProt IDs), mutations, and optional inputs for Tm and pH. If pH and Tm values are not provided, the program defaults to pH 7.0 and utilizes ProtStab 2.0 [24] predictions for Tm. Additionally, users have the choice to use the Common version of PON-Tm, which obviates the need for entering any extra conditions. Once the process is finished, the initiator receives a detailed results report via the specified email address. Both the training and testing datasets, as well as MutDB, are freely downloadable from the website.

## 3. Discussion

Machine-learning-based methods have gained widespread application in predicting the impact of mutations on protein thermal stability, primarily due to the time-consuming and labor-intensive nature of experimental measurements. The significance of mutations in protein engineering lies in their potential to create proteins capable of maintaining activity and functionality in environments far beyond their physiological temperature range. This has profound implications for various fields, including biopharmaceuticals, enzyme engineering, and food engineering. To this end, we have developed a predictor PON-Tm solely reliant on protein sequences, with the added flexibility to accommodate environmental conditions, thus enhancing the precision and versatility of mutation impact predictions.

We firstly constructed MutDB by integrating data from three distinct sources, incorporating environmental conditions pH and Tm. Our findings revealed that incorporating both pH and Tm into predictions yields superior results compared to considering only Tm or neglecting environmental conditions altogether. Moreover, currently, the available datasets we have utilized all focus on missense mutations, which led to our initial version of PON-Tm being tailored for missense mutations.

Protein embeddings are a critical component of sequence-based predictors. They transform protein sequences into a high-dimensional space where biologically relevant features are captured. In our study, we employed three protein language models, namely ProtBert, ESM-2-650M, and ESM-2-3B, for protein embedding. ProtBert and ESM-2-650M are both state-of-the-art models, but ESM-2-3B stands out due to its larger training dataset and more sophisticated architecture [14], which likely contributes to its superior performance. This larger model size allows ESM-2-3B to capture more complex patterns in protein sequences, which is crucial for accurate mutation impact prediction.

Furthermore, we investigated the impact of context length on model efficacy, specifically utilizing ESM-2-3B as the embedding tool. We conducted tests using six different context lengths: 25, 50, 100, 200, 500, and the full protein sequence length. The findings revealed a general trend of improved model performance with increased context length, peaking when the context length was set to 500. However, this longer context length also demands a higher quality of predicted protein sequences and carries a greater risk of overfitting. Consequently, we opted for a more balanced approach, selecting a context length of 200 for subsequent training sessions. It is noteworthy that utilizing the entire protein sequence length did not yield optimal performance. This unexpected result may stem from two factors. Firstly, longer sequences may introduce extraneous and irrelevant information, obscuring the pertinent signal for mutation impact prediction. Secondly, the full sequence could encompass biologically irrelevant regions relative to the mutation site, thus weakening the predictive signal.

However, several considerations must be taken into account for future research directions. Primarily, while pH and Tm have been shown to significantly influence protein stability, the protein universe is far more complex, and other environmental conditions undoubtedly play a role. For instance, ion concentrations, which can greatly affect protein folding and stability, were not included in our current model. In the next iteration of PON-Tm, it would be prudent to expand the environmental parameter set to encompass a broader range of conditions. This would not only enhance the model’s predictive accuracy but also broaden its applicability to a wider array of biological contexts. Moreover, another significant challenge we currently face is the limited availability of data and a single mutation type (just missense mutations). The construction of effective and robust models, especially those utilizing advanced techniques like deep learning, heavily relies on extensive and diverse datasets. Without sufficient data, these models may struggle to generalize well, leading to potential overfitting or underfitting issues. Future efforts should focus on expanding the available datasets through collaborations or data sharing initiatives. As data accumulate, we intend to refine PON-Tm, extending its functionality to handle various mutation types, including deletions or duplications. This advancement will widen our server’s applicability, facilitating a more in-depth mutation analysis. Additionally, researchers could explore alternative modeling approaches that are more data-efficient, such as transfer learning or semi-supervised learning, which can leverage unlabeled data or knowledge transfer from related tasks.

## 4. Materials and Methods

### 4.1. Datasets

**Figure 3:**
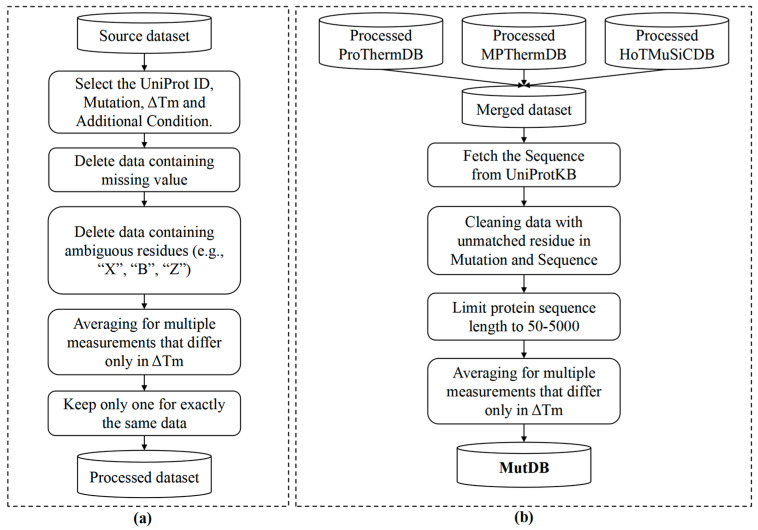
The procedure of MutDB construction. (**a**) Procedure for each dataset; (**b**) combination of the data from ProThermDB, HoTMuSiC, and MPTherm.

We systematically organized the collected mutations based on varying environmental conditions and constructed the MutDB dataset by integrating datasets from ProThermDB [15], HoTMuSiCDB, and MPThermDB [18]. Each dataset underwent a rigorous cleansing process (Figure 3a) before being merged to create a unified version of MutDB (Figure 3b). Specifically, ProThermDB represents an updated thermodynamic database (https://web.iitm.ac.in/bioinfo2/prothermdb/index.html, accessed on 11 March 2024) for proteins and their mutants, encompassing approximately 20,000 data points on protein thermostability changes determined through recent high-throughput proteomics techniques using whole-cell approaches. HoTMuSiCDB, on the other hand, serves as the benchmark dataset for HotMuSiC [16,17], with its data primarily sourced from the literature (70%) and supplemented by data from ProThermDB (30%) [25]. MPThermDB was carefully selected from MPTherm V1.0 (https://www.iitm.ac.in/bioinfo/mptherm/, accessed on 11 March 2024), a valuable resource for understanding the thermostability changes of membrane proteins. Additionally, since our PON-Tm relies solely on protein sequences, to ensure fair comparisons among different predictors, we randomly selected 10% of the mutations from MutDB-Common as a test set, denoted as T267, where both PDB ID and UniProt ID can be clearly matched. The remaining data from MutDB were designated as the training set for model development.

### 4.2. Traditional Features

We sourced 349 sequences from UniProtKB [26], ultimately deriving 1143 features to comprehensively describe protein characteristics.

Amino Acid Properties: AAindex (https://www.genome.jp/aaindex/, accessed on 11 March 2024) [27] comprises 685 physicochemical and biochemical properties of amino acids. After the elimination of entries with missing values, 617 properties remain. Specifically, AAindex1 details the amino acid index of 20 numerical values, AAindex2 outlines the amino acid mutation matrix, and AAindex3 defines statistical protein contact potentials. For each mutation, we calculate the difference in AAindex1 values between the wild and mutant amino acids to generate features, while directly obtaining additional features from AAindex2 and AAindex3.

Neighborhood Characteristics: We postulate that sequences within a limited length of 25 amino acids around the mutation site significantly impact the mutation’s value, whereas residues beyond this range exert minimal influence. To capture these effects, we compute the frequency of each amino acid type within this 25-residue window, yielding 20 features corresponding to the common amino acids [28].

Amino Acid Grouping: Amino acids can be categorized into six groups based on their clusterable physicochemical properties: hydrophobic (V, I, L, F, M, W, Y, C), negatively charged (D, E), positively charged (R, K, H), conformational (G, P), polar (N, Q, S), and others (A, T). We employed a 6 × 6 matrix to systematically represent the alterations in properties associated with the transition from wild-type to mutant amino acids. By extending this matrix, we derived 36 distinct features that comprehensively capture the variations [29].

Positional Features: The Position-Specific Scoring Matrix (PSSM) reflects the conservation level at each protein sequence position, indicating the likelihood of an amino acid remaining unchanged across homologous sequences. We generate the PSSM using PSI-BLAST, configured with 3 iterations and an E-value cutoff of 1E-3. To standardize feature representation across sequences of varying lengths, we divide the original PSSM into 20 sections and use the average of each column to represent each section, resulting in a total of 400 features [30,31].

Physicochemical Properties: ProtParam (https://web.expasy.org/protparam/, accessed on 5 April 2024) [32] was used to calculate various physicochemical properties from sequences. We selected the seven most pertinent features for protein thermal stability: the number of amino acids, molecular weight, theoretical pI, total number of atoms, extinction coefficient, instability index (II), and aliphatic index.

Evolutionary Conservation: The SIFT4G algorithm [33] predicts the deleteriousness of amino acid substitutions, bridging the gap between mutations and phenotypic variations. SIFT4G outperforms SIFT due to its expanded database and GPU assistance. We obtained SIFT4G from https://github.com/rvaser/sift4g (accessed on 5 April 2024) and computed three features: SIFT_SCORE, SIFT_MEDIAN, and NUM_SEQ.

Scale Features: ProtScale [32] was employed to compute profiles generated by any amino acid scale on a chosen protein, offering 57 predefined scales from the literature. We sourced values from https://web.expasy.org/protscale/and (accessed on 5 April 2024) derived 57 features for each mutation by calculating the difference between mutant and wild residue scales.

### 4.3. Protein Embedding

**Figure 4:**
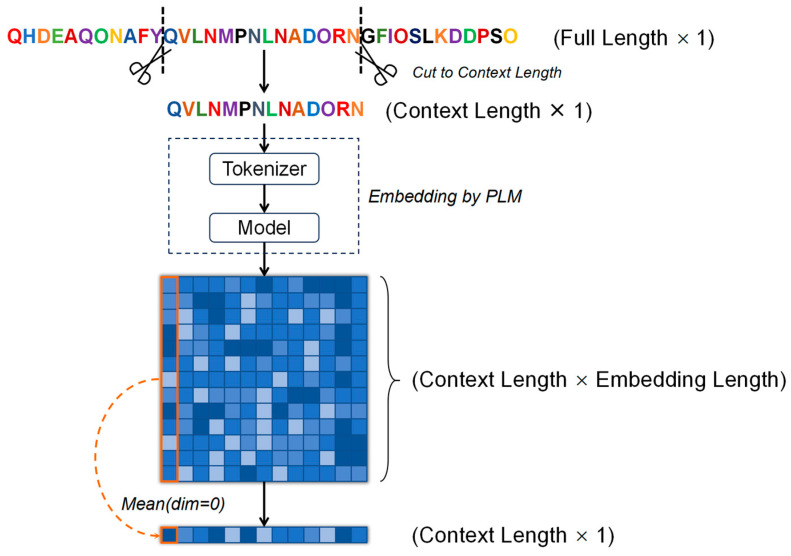
Protein embedding process.

In recent years, significant advancements have been made in the field of natural language processing due to deep learning, particularly in the domain of transfer learning. By analogy, we consider amino acids in protein sequences as equivalent to letters in natural language, enabling the application of transfer learning techniques to proteins. This methodology provides valuable insights into the characteristics of protein sequences. In our study, we utilized ESM-2 [14] and ProtBert [22] to extract embedded features from protein sequences (Figure 4). Specifically, we employed two versions of ESM-2, namely ESM-2-3B and ESM-2-650M, distinguished by their parameter counts, where a higher number of parameters generally correlates with enhanced prediction reliability.

As shown in Figure 4, the initial protein sequence length is denoted as Full Length. A specified context sequence length, designated as Context Length, is then determined around the mutation site. Sequences with a Full Length shorter than the designated Context Length are excluded from analysis. The sequence is subsequently processed through a Tokenizer to generate corresponding tokens, which are then fed into the model to produce a feature matrix of dimensions [Context Length, Embedding Length]. By applying a Mean operation with dim set to 0, we obtain a feature vector of shape [Context Length,] that characterizes the mutation context structure. If the entire sequence, rather than a contextual subsequence, is used (i.e., Full Length equals Context Length), the feature matrix must be transposed, resulting in a feature vector of shape [Embedding Length,]. Differences in model architectures, such as those between ESM-2 and ProtBert, influence parameter counts, Embedding Length, and the model’s protein representation capabilities (Table 9). Additionally, during feature extraction, it is essential to obtain embeddings for both wild-type and mutant sequences, yielding two feature vectors of the same length.

### 4.4. Feature Selection and Regression Algorithms

In our study, we utilized RFE and RFECV for feature selection. RFE iteratively selects features based on an external estimator, taking into account the assigned feature weights. Initially, the estimator is trained using the available features, and the importance of each feature is determined. Subsequently, the least significant features are eliminated. This iterative pruning process continues until the desired number of features is reached. On the other hand, RFECV integrates RFE (sckit-learn package version 1.4.1, accessed on 24 April 2024 from https://scikit-learn.org) into a cross-validation framework to ascertain the optimal number of features. Specifically, we employed 5-fold cross-validation within RFECV for our analysis.

For the ultimate prediction model development, we concurrently utilized two distinct regression algorithms, namely LightGBM [34] and XGBoost [35], aiming to achieve optimal results. LightGBM (version 4.3.0, accessed on 1 March 2024 from https://github.com/microsoft/LightGBM) is a gradient-boosting framework that relies on tree-based learning algorithms. Meanwhile, XGBoost (version 2.0.3, accessed on 1 March 2024 from https://github.com/dmlc/xgboost) stands as an optimized distributed gradient-boosting library and uses a parallel tree boosting.

Both algorithms were executed in Python with their default parameters, ensuring consistency in our evaluation. The entire scripting process was conducted using Python 3.11.9.

### 4.5. Evaluation Metrics

Given that our protein thermostability change prediction constitutes a regression problem, we employ four metrics for a comprehensive evaluation of the prediction model: the Pearson correlation coefficient (*PCC*), coefficient of determination (*R*^2^), root mean square error (RMSE), and mean absolute error (*MAE*).

The *PCC*, ranging from −1 to 1, quantifies the linear relationship between two variables. It is calculated by dividing the covariance of the two variables by the product of their standard deviations. The *PCC* provides a measure of the strength and direction of the linear association between the variables. A higher absolute value of the *PCC* signifies a stronger linear correlation. Mathematically, it is expressed as follows:

(1) $$\begin{array}{c} PCC=\frac{\mathrm{cov}\left(X,Y\right)}{\sigma_{X}\sigma_{Y}}=\frac{E\left(XY)-E(X\right)E\left(Y\right)}{\sqrt{E\left(X^{2}\right)-E^{2}\left(X\right)}\sqrt{E\left(Y^{2}\right)-E^{2}\left(Y\right)}} \end{array}$$

where cov is the covariance, $\sigma_{X}$ is the standard deviation of X, $\sigma_{Y}$ is the standard deviation of Y, and E is the mathematical expectation.

The coefficient of determination, denoted as *R*^2^, represents the proportion of the variance in the dependent variable that can be predicted from the independent variable(s). In the context of regression analysis, it assesses the model’s goodness of fit, indicating how well the model explains the variation in the dependent variable. An *R*^2^ value closer to 1 signifies a better fit of the model to the data. However, it is important to note that in certain circumstances, such as when the model’s predictions are worse than simply using the mean of the dependent variable, the adjusted *R*^2^ can even be negative.

(2) $$\begin{array}{c} R^{2}=1-\frac{{SS}_{res}}{{SS}_{tot}}=1-\frac{\sum_{i=1}^{m}{\left(y_{i}-{\hat{y}}_{i}\right)}^{2}}{\sum_{i=1}^{m}(y_{i}-\bar{y}{)}^{2}} \end{array}$$

where ${SS}_{tot}$ is the total sum of squares, ${SS}_{res}$ is the sum of squares of residuals. ${\hat{y}}_{i}$ is the experimental value, $y_{i}\;$is the predicted value, and $\bar{y}\;$is the average of all predicted values.

*RMSE* measures the deviations between predicted and observed values:

(3) $$\begin{array}{c} RMSE=\sqrt{\frac{1}{m}\sum_{i=1}^{m}{\left(y_{i}-{\hat{y}}_{i}\right)}^{2}} \end{array}$$

*MAE* represents the average of the absolute differences between predicted and observed values. As a non-negative real number, MAE offers a direct measure of prediction accuracy, where a lower MAE signifies a more accurate model.

(4) $$\begin{array}{c} MAE=\frac{1}{m}\sum_{i=1}^{m}\left|y_{i}-{\hat{y}}_{i}\right| \end{array}$$

where ${\hat{y}}_{i}$ is the experimental value and $y_{i}\;$is the predicted value.

## 5. Conclusions

In this study, we introduced PON-Tm, an advanced method for predicting missense-mutation-induced changes in protein thermal stability. By integrating sequence features from a protein language model with environment-dependent factors like pH and wild-type thermostability, PON-Tm enhances prediction accuracy.

## Figures and Tables

**Table 1:** Three versions of MutDB and corresponding sub-datasets.

Sub-Datasets	Environment-Dependent Condition	Number	Variance of ΔTm
MutDB-Common	None	3439	84.08
ProThermDB-Common		2196	
HoTMuSiCDB-Common		1199	
MPThermDB-Common		853	
MutDB-Tm	Tm	7057	56.32
ProThermDB-Tm		4572	
HoTMuSiCDB-Tm		1129	
MPThermDB-Tm		1704	
MutDB-pH-Tm	pH, Tm	7076	56.41
ProThermDB-pH-Tm		4595	
HoTMuSiCDB-pH-Tm		1137	
MPThermDB-pH-Tm		1712	

**Table 2:** Information of Iso-1 cytochrome c protein with K79I mutation.

pH	Tm (°C)	ΔTm (°C)
3.0	22.3	0.8
3.5	31.7	1
4.0	38.7	−2
4.5	45.2	0.2
5.0	47.7	−5

**Table 3:** Model performance under different environmental factors.

Dataset	MAE (°C)	RMSE (°C)	R^2^	PCC
MutDB-Common	4.312	7.068	0.271	0.563
MutDB-Tm	2.134	3.755	0.752	0.868
MutDB-pH-Tm	**2.030**	**3.602**	**0.774**	**0.881**

Bold signifies the most favorable result.

**Table 4:** Performance on 10-fold cross-validation using different embedding models.

Model	MAE (°C)	RMSE (°C)	R^2^	PCC
ProtBert	2.044	3.592	0.774	0.881
ESM-2-650M	2.030	3.602	0.774	0.881
ESM-2-3B	**2.014**	**3.572**	**0.778**	**0.883**

Bold signifies the most favorable result.

**Table 5:** Performance on 10-fold cross-validation with different context lengths.

Length	MAE (°C)	RMSE (°C)	R^2^	PCC
25	2.215	3.583	0.772	0.879
50	2.218	3.617	0.769	0.877
100	2.153	**3.525**	0.770	0.879
200	**2.039**	3.602	0.774	0.881
500	2.182	3.770	**0.810**	**0.902**
full-length	2.163	3.574	0.774	0.880

Bold signifies the most favorable result.

**Table 6:** Results for feature selection with RFE and RFECV.

Performance	RFE-50	RFE-100	RFE-200	RFE-500	RFE-1000	RFE-3704	RFECV-595
MAE (°C)	**1.828**	1.868	1.930	1.961	1.955	2.093	1.935
RMSE (°C)	3.744	3.526	**3.420**	3.465	3.516	3.868	3.556
R^2^	0.786	0.792	**0.797**	0.784	0.757	0.743	0.794
PCC	0.887	0.891	**0.893**	0.886	0.871	0.865	0.892

Bold signifies the most favorable result.

**Table 7:** Two hundred features selected by RFE.

Feature	Number	Feature	Number
pH	1	Param	5
Tm	1	Pssm	59
Neighbor	3	Scale	10
AAindex	64	Embedding	57

**Table 8:** Performance comparison on T267.

Tool	MAE (°C)	RMSE (°C)	R^2^	PCC
AUTOMUTE_SVM	4.777	7.061	0.275	0.535
AUTOMUTE_REPT	5.568	7.862	0.102	0.378
HoTMuSiC	4.747	7.363	0.264	0.564
MPTherm-pred	7.069	9.330	−0.191	0.176
PON-Tm	**4.253**	**6.105**	**0.477**	**0.695**

Bold signifies the most favorable result.

**Table 9:** The number of parameters and Embedding Length of the three models.

Model	Number of Parameters	Embedding Length
ProtBert	420M	1024
ESM-2-650M	650M	1280
ESM-2-3B	3B	2560

## Data Availability

The data used in this paper are available at https://www.yanglab-mi.org.cn/Pon-Tm.

## Supplementary Materials

The following supporting information can be downloaded at https://www.mdpi.com/article/10.3390/ijms25158379/s1.
